# Insertion Sequence (IS) Element-Mediated Activating Mutations of the Cryptic Aromatic β-Glucoside Utilization (*BglGFB*) Operon Are Promoted by the Anti-Terminator Protein (BglG) in *Escherichia coli*

**DOI:** 10.3390/ijms23031505

**Published:** 2022-01-28

**Authors:** Zhongge Zhang, Kingswell Zhou, Dennis Tran, Milton Saier

**Affiliations:** Division of Biological Sciences, Department of Molecular Biology, University of California, La Jolla, CA 92093-0116, USA; zzhongge@ucsd.edu (Z.Z.); kiz001@ucsd.edu (K.Z.); det005@ucsd.edu (D.T.)

**Keywords:** transposon, insertion sequences, *bglGFB* operon, BglG anti-terminator, stress induced mutation, directed mutation, SIDD structure

## Abstract

The cryptic β-glucoside GFB (*bglGFB*) operon in *Escherichia coli* (*E. coli*) can be activated by mutations arising under starvation conditions in the presence of an aromatic β-glucoside. This may involve the insertion of an insertion sequence (IS) element into a “stress-induced DNA duplex destabilization” (SIDD) region upstream of the operon promoter, although other types of mutations can also activate the *bgl* operon. Here, we show that increased expression of the *bglG* gene, encoding a well-characterized transcriptional antiterminator, dramatically increases the frequency of both IS-mediated and IS-independent Bgl^+^ mutations occurring on salicin- and arbutin-containing agar plates. Both mutation rates increased with increasing levels of *bglG* expression but IS-mediated mutations were more prevalent at lower BglG levels. Mutations depended on the presence of both BglG and an aromatic β-glucoside, and *bglG* expression did not influence IS insertion in other IS-activated operons tested. The N-terminal mRNA-binding domain of BglG was essential for mutational activation, and alteration of BglG’s binding site in the mRNA nearly abolished Bgl^+^ mutant appearances. Increased *bglG* expression promoted residual *bgl* operon expression in parallel with the increases in mutation rates. Possible mechanisms are proposed explaining how BglG enhances the frequencies of *bgl* operon activating mutations.

## 1. Introduction

“Directed mutation” is defined as a biological process or phenomenon whereby favorable mutations occur at higher frequencies than neutral or deleterious mutations in response to a stressful environmental condition, where the mutations that arise specifically minimize the deleterious organismal response to the stress [1,2]. Thus, it has been proposed that an environmental pressure can cause advantageous mutations to occur with increased frequencies in specific genes/operons to alleviate that particular environmental stressful pressure [3,4]. Directed mutation, as proposed by Cairns and defined above, may be an exception to the generally accepted rule that “mutations arise randomly, and the advantageous ones are subsequently selected for” [5]. Although the process of directed mutation has never been disproven, and convincing evidence in favor of its occurrence has been published, the concept is generally in disfavor among geneticists [6].

Several researchers have suggested that directed mutation does occur in nature, although relatively few examples have been extensively documented. The first and perhaps best documented example, in which numerous control experiments have been performed to verify the occurrence of directed mutation, involved transposon Insertion Sequence 5 (IS5)-mediated activation of the *Escherichia coli (E. coli) glycerol FK (glpFK)* operon [7,8]. However, Wang and Wood subsequently described an example of IS-activation of the *flagella master switch (flhDC)* operon encoding the master regulator of motility in *E. coli* [9], confirmed and extended in a later study by our research group [10]. Another example deals with transposon-activation of zinc resistance in *Cupriavidus metallidurans* in a mechanistically fairly well-defined process [11]. Two other examples involve transposon excision that seems to be regulated [5,12]. It appears that such examples are not uncommon in both bacteria and eukaryotes [13]. Additional examples in bacteria include IS5 activation of the *fucose AO (fucAO)* operon in *E. coli* [14], *furazolidone sensitivity A* and *B* (*nfsA* and *nfsB)* inactivation by IS elements, also in *E. coli* [15,16], the regulation of capsule biosynthesis in *Neisseria meningitidis* [17], developmentally programmed transposon excision in the *Bacillus subtilis* sporulation cascade [12], drug (piperacillin/tazobactam) resistance in *E. coli* [18], and retrotransposon-regulated gene expression in eukaryotes [19,20]. Thus, there is an ever-increasing number of examples of the regulation of gene expression and function that may be subject to transposon-mediated directed or adaptive mutation. It should be noted, however, that most of the IS-regulated gene expression studies described above have not been mechanistically defined.

**Scheme 1:**
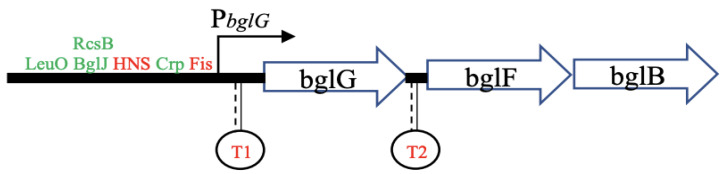
The *bgl* operon.

We have used *E. coli* as our model organism for studying the involvement of transposons in mediating environmentally promoted, DNA binding protein-controlled, gene/operon activation with examples as noted above. In this report we turn to a well characterized operon, the *β-glucoside GFB (bglGFB)* operon of *E. coli*, that has been known for decades to be activated by IS insertional events [21,22]. Although great effort has been devoted to understanding the transcriptional regulation of *bgl* operon expression by an antitermination mechanism, very little research has described the mechanism(s) by which IS insertional mutations of the *bgl* operon are regulated Scheme 1.

**Figure 1:**
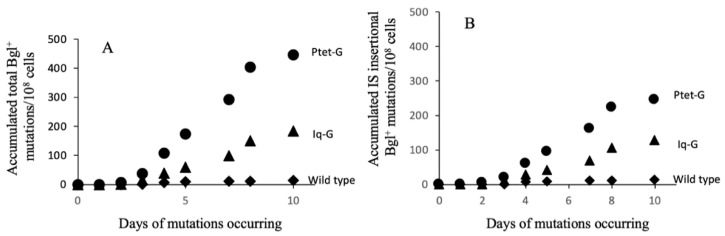
Effects of different levels of constitutively expressed *bglG* on Bgl^+^ mutations on minimal M9 + salicin agar plates. A total of 2 × 10^7^ cells were applied onto each minimal M9 agar plate containing 0.5% salicin as the sole carbon source. The plates were incubated at 30 °C, being examined daily for the appearance of Bgl^+^ colonies. The total Bgl^−^ cell populations were determined as reported previously [8,31]. The frequencies of Bgl^+^ mutations were determined by dividing the number of Bgl^+^ colonies by the total Bgl^−^ populations. (**A**) Accumulated total Bgl^+^ mutations in cells constitutively expressing *bglG*. A new copy of *bglG*, driven by the strong constitutive promoter P*tet* (strain Ptet-G) or the weaker promoter *lacIq* (strain Iq-G), was located at the *intS* locus on the chromosome. Total Bgl^+^ mutations include IS insertional mutations and non-IS insertional mutations. (**B**) Accumulated IS insertional Bgl^+^ mutations in cells constitutively expressing *bglG*. At least 100 independent Bgl^+^ mutants for each test strain were used to determine the ratio of IS insertional mutants versus non-insertional mutants. Note: The *x*-axis shows the day that mutations occurred, not when mutant colonies become visible. The same use of this *x*-axis occurs in Figure 1, Figure 2, Figure 3, Figure 4, Figure 5 and Figure 6 where applicable (see Materials and Methods).

**Figure 2:**
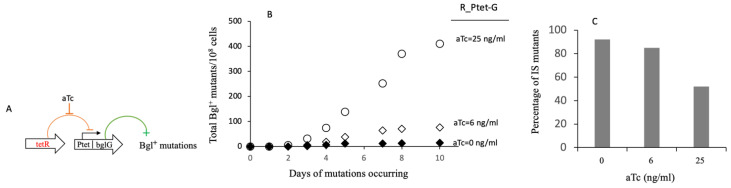
Dependence of total Bgl^+^ mutations and IS insertional Bgl^+^ mutations on BglG levels. A total of 2 × 10^7^ cells of strain R_Ptet-G were applied on M9 + salicin agar plates with various levels of aTc (the inducer for TetR-repressed P*tet*). The Bgl^+^ mutations were carried out as for Figure 1. (**A**) Diagram showing TetR inhibition of *bglG* expression and BglG activation of Bgl^+^ mutations. As an inducer, aTc releases TetR from the P*tet* promoter, thereby allowing *bglG* expression. The more aTc added, the greater the expression of *bglG*. In the absence of an inducer (aTc), no additional BglG (BglG production is only from the native *bgl* operon) is produced. (**B**) Effects of titrated *bglG* expression on total Bgl^+^ mutations. (**C**) Effects of BglG levels on the ratios of IS insertional mutants out of the total number of Bgl^+^ mutants.

As noted above, increased expression of the *bglG* gene increases both IS-insertional mutations and non-IS-insertion mutations, although these two types of mutations do not arise in parallel as a function of BglG levels (see Figure 1 and Figure 2 in the Section 2). Regarding the nature(s) of the non-IS insertional mutants (which are not the focus of this paper), several investigators have considered their identities in the past, and their published results suggest that mutations in several genes within and outside of the *bglGFB* operon on the *E. coli* chromosome may be responsible for a Bgl^+^ phenotype when starting with a wild-type strain that is cryptic for *bgl* operon expression. Below, we list some of these published results.

(1) A point mutation in the cyclic AMP (cAMP) Receptor Protein (Crp)-cAMP operator, leading to a better binding site for the Crp-cAMP complex, can lead to enhancement of *bgl* operon expression [21]. (2) Increased expression of either of two genes outside of the *bgl* operon, *bglJ* and DNA-binding transcriptional dual regulator *leuO*, can activate *bgl* operon expression [23,24]. (3) DNA supercoiling, determined primarily by the relative activities of topoisomerase I and gyrase (topoisomerase II; *gyrA*/*gyrB*) influences *bgl* operon expression [25,26,27]. (4) Loss of the histone-like nucleoid protein (H-NS) activates the cryptic *bgl* operon, revealing that H-NS is a primary repressor of *bgl* operon expression [28]. (5) The Lon protease and the RNA-binding protein, Hfq, reduce silencing of the *E. coli bgl* operon by H-NS [29]. (6) The DnaJK chaperone pair strengthens H-NS-mediated repression of the *bgl* operon (increasing H-NS binding within the *bglG* gene), so that defective DnaJK function yields increased *bgl* operon expression [30]. These observations suggest that several types of mutations, not involving transposon insertion upstream of the *bgl* operon promoter, can lead to increased expression of the cryptic *bgl* operon in wild type *E. coli* cells. The fact that these mutations (or at least some of them) increase with increased *bglG* expression as demonstrated here is noteworthy and may suggest that at least some types of mutations, not characterized to date, may be under the control of BglG and may therefore be “directed”.

This paper is the first of our systematic reports aimed at the characterization of this process. We show that the known anti-terminator protein, BglG, is essential for the appearance of Bgl^+^ mutations (Bgl^+^ here defined as a cell’s ability to grow on a β-glucoside as the sole carbon source). BglG is a specific positive regulator of the appearance of IS insertional mutations as well as of other types of uncharacterized mutations that promote *bgl* operon activation, apparently by IS-independent but poorly defined processes. We further provide evidence that the *bgl* operon insertional process is dependent on environmental conditions and only occurs in the presence of an aromatic β-glucoside substrate of the sugar catabolic system encoded by the *bglGFB* operon. Possible mechanisms are proposed.

## 2. Results

### 2.1. Overexpression of the BglG Gene Increases the Frequencies of Both IS- and Non-IS-Mediated Bgl^+^ Mutations

Figure 1A shows the total numbers of Bgl^+^ mutations arising on salicin M9 minimal agar plates as a function of time in wild type *E. coli* cells as well as the same cells in which *bglG* was expressed with the use of the intermediate lactose operon repressor promoter P*lacIQ* (strain Iq-G) or the stronger tetracycline promoter P*tet* (strain Ptet-G). These promoters drive *bglG* expression to moderate and high levels, respectively. Figure 1A reveals that the accumulated total number of Bgl^+^ mutations increased many folds following increased expression of *bglG*, and the stronger the promoter, the higher the frequency of mutations. After 12 days of incubation, the total mutation frequencies, expressed in terms of total mutants per total cell numbers, increased 14- and 33-fold in response to *bglG* expression driven by *lacIq* and P*tet*, respectively. These experiments were repeated multiple times on salicin M9-agar plates, and similar results were attained.

Bgl^+^ mutation assays were also conducted on arbutin M9 minimal agar plates, comparing the wild type and the *bglG* overexpression strain Ptet-G (Figure S1). Up to 20-fold more Bgl^+^ mutants arose from strain Ptet-G compared to the wild type strain, although overall fewer mutations occurred on arbutin agar plates than on salicin plates, possibly due to the production of a toxic compound released by arbutin hydrolysis. Among all the Bgl^+^ mutants, about 53% were IS insertional mutants. 

The total Bgl^−^ populations (background populations) on M9 + salicin agar plates were determined over time (see Materials and Methods) as summarized in Figure S2. For all three test strains (wild type, Iq-G and Ptet-G), slight increases (about 1.5 generations) of total background populations were observed during the first two days after plating. This may be due to the use of some trace nutrients present in the agar media. Afterwards, the Bgl^−^ background populations were maintained relatively constantly (about 6 × 10^7^ CFU per plate) for all test strains. 

We also tested the growth of these strains in liquid M9 minimal medium with salicin (0.5%) as the sole carbon source. As seen in Figure S3, over a 45 h incubation period with shaking at 30 °C, no obvious growth was observed for these three strains, confirming that increased expression of *bglG* using a single copy of a chromosomal cassette did not affect the Bgl^−^ population appreciably.

As noted in the Introduction, Bgl^+^ mutations consist of IS insertional and IS-independent mutations. These types of mutations can be distinguished using colony Polymerase Chain Reaction (PCR). Figure 1B shows IS insertional Bgl^+^ mutations as a function of time for the same three strains used in Figure 1A. Of >100 independent Bgl^+^ mutants derived from each strain tested, about 95%, 70% and 55% are due to IS insertions upstream of the native *bgl* operon promoter in wild type, Iq-G and Ptet-G cells, respectively. As compared to the wild type cells, IS-insertional mutations increased about 10- and 20-fold in Iq-G and Ptet-G cells, respectively (compare Figure 1A,B).

Sequencing analyses showed that most (>90%) of the insertional mutants have an IS1 or IS5 element inserted upstream of the *bgl* operon promoter while the rest have IS2 or IS10 insertions (data not shown). Among IS-independent Bgl^+^ mutants, a small fraction (<5%) have a duplication of 12 nucleotides in the *hns* gene (see bolded sequences and red-faced sequences in Figure S4A). Such a duplication mutation led to the duplication of four residues “EMLE” (bolded residues in Figure S4B) in the encoded protein. After being transferred to wild type cells via P1 transduction, these mutants were capable of growth on salicin as the sole carbon source, indicating that this duplication mutation in *hns* is sufficient to cause a Bgl^+^ phenotype without causing a large growth defect. However, for most IS-independent Bgl^+^ mutants, the gene targets of the responsible mutations have not been identified. No mutation was identified in the known targets described in the Introduction, including the Crp-cAMP operator upstream of the *bgl* promoter and the structural genes or the regulatory regions of *bglJ*, *leuO*, *hns*, *gyrA*/*gyrB*, *DnaK*, *lon* and *hfq*.

Figure 2 shows the consequences of differential induction of *bglG* expression on mutation frequencies. In Figure 2A, the strategy used for controlling the level of *bglG* expression in strain R_Ptet-G is illustrated. In this strain, the constitutive expression of the Tet repressor gene, *tetR*, inhibits expression of *bglG* behind P*tet*. In the absence of the inducer, anhydrous tetracycline (aTc), almost no additional *bglG* expression was observed over the native level. Regardless of the level of *bglG* expression, mutations to a Bgl^+^ phenotype arose. The frequencies of Bgl^+^ mutations increased roughly proportional to the level of *bglG* expression, which in turn was proportional to the amount of aTc present in the media (Figure 2B). Colonies from salicin plates (Figure 2B) were picked, purified and identified by colony PCR for IS insertions. Although both the total numbers of Bgl^+^ mutations and of IS-insertional Bgl^+^ mutations increased with the aTc inducer concentrations, the percentages of IS-insertional mutations decreased with increasing amounts of aTc present (Figure 2C). Thus, the non-IS insertional mutants increased in number more than IS insertional mutants as the level of *bglG* expression increased. This is consistent with the results described in Figure 1.

To further show *bglG* expression is required for Bgl^+^ mutations, strain G85stop (Figure S5A; Table S1) was made, in which 28 codons in the 5′ end of *bglG* were replaced by 27 different codons plus a stop codon. This prevented functional BglG production from the modified *bglG* gene. The constitutively expressed *tetR* and the P*tet*-*bglG* cassette were transferred to G85stop, yielding strain R_G85stop_Ptet-G. Due to the high level of TetR (the *tet* repressor), no BglG protein was produced from R_G85stop_Ptet-G cells in the absence of inducers. This strain was subject to Bgl^+^ mutation assays on M9 + salicin agar plates with various amounts of aTc. As seen in Figure S5B, no mutations were observed in the absence of aTc, confirming that BglG is essential for Bgl^+^ mutations. When aTc was added to the test media, Bgl^+^ mutants arose with more aTc inducer, leading to more mutants. However, the mutation frequencies were much lower than ones observed for strain R_Ptet-G which carries the native *bglG* in the *bgl* operon (Figure 2B and Figure S5B). 

### 2.2. Appearance of Bgl^+^ Mutations Depends on the Presence of a β-Glucoside

**Figure 3:**
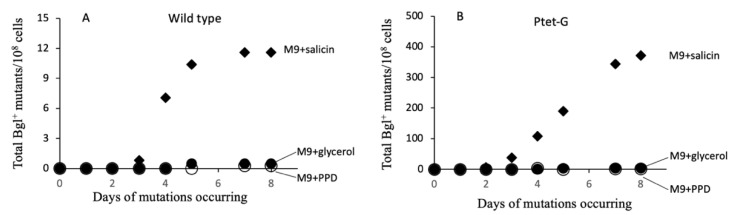
Dependence of Bgl^+^ mutations on b-glucoside. (**A**) Effects of salicin, glycerol and propandiol on Bgl^+^ mutations in wild type cells. (**B**) Effects of salicin, glycerol and propandiol on Bgl^+^ mutations in Ptet-G cells expressing *bglG*. In (**A**,**B**), 2 × 10^7^ cells were applied to M9 + salicin or glycerol plates while 10^8^ cells were applied to M9 + PPD plates. The plates were incubated at 30 °C. For M9 + salicin plates, the Bgl^+^ mutation frequencies were determined as in Figure 1. For M9 + glycerol or PPD plates, the cells were washed off the agar plates and diluted with M9 salts. Appropriate dilutions were applied to LB plates to determine the total cell populations, and to M9 + salicin plates to determine the Bgl^+^ populations. The Bgl^+^ mutation frequencies were determined by dividing the Bgl^+^ populations by the total populations.

Figure 3 shows the appearance of Bgl^+^ mutations in the presence of various carbon sources present in the minimal M9 agar plates. These sources included the aromatic β-glucoside, salicin, as well as glycerol and propanediol (PPD). For salicin agar plates, the Bgl^+^ mutation frequencies were determined as described in Figure 1 and Figure 2. For glycerol or PPD agar plates, at various time intervals after plating, cells from 1 cm wide cores, obtained from each plate where no colonies appeared, were washed off the cores with M9 salts (with 90% retention of cells). Cell suspensions were properly diluted as required and applied to LB agar plates (for total cell counts) and to minimal (M9) plates with salicin as the sole carbon source (for measurement of Bgl^+^ mutant cells). As seen in Figure 3A, when only wild type levels of BglG were present, essentially no Bgl^+^ mutations arose in the absence of a β-glucoside (that is, glycerol or PPD plates), but in the presence of salicin, Bgl^+^ mutants appeared. When a second copy of the *bglG* gene was expressed behind the P*tet* promoter, many more Bgl^+^ colonies appeared on salicin agar plates as expected (Figure 3B). On the other hand, even with high levels of BglG, no Bgl^+^ mutants appeared in the absence of a β-glucoside. Thus, in both Figure 3A (with wild type levels of BglG) and Figure 3B (with high levels of BglG), mutation rates dramatically increased only when a β-glucoside was present. 

### 2.3. Increases in the Frequencies of Bgl^+^ Mutations Due to the Overexpression of the BglG Gene Are Bgl Operon Specific

**Figure 4:**
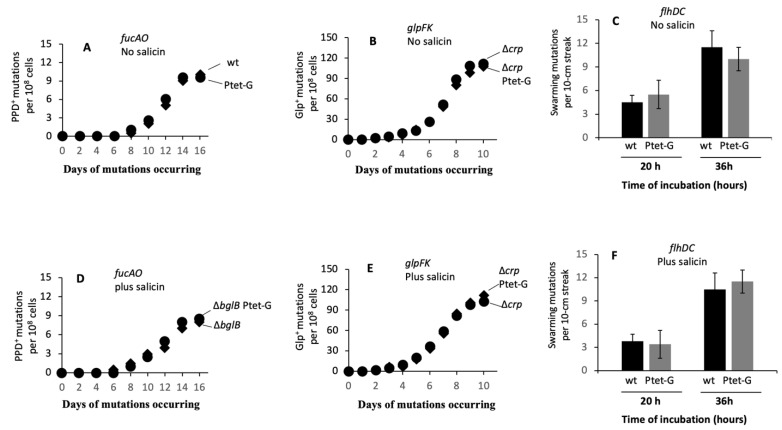
Increased expression of *bglG* affects only IS insertions into the *bgl* operon but not the *fucAO*, *glpFK* and *flhDC* operons. (**A**) Effect of *bglG* expression on PPD^+^ mutations. A total of 10^8^ cells were applied onto M9 + PPD plates, and PPD^+^ colonies were counted over time. (**B**) Effect of *bglG* expression on Glp^+^ mutations. A total of 2 × 10^7^ cells of a *crp* deletion strain with or without P*tet* driving *bglG* were applied onto M9 + glycerol plates, and Glp^+^ colonies were counted over time. (**C**) Effect of *bglG* expression on swarming mutations. A total of 2 × 2 μL of cell suspensions with an OD_600_ of 1 were streaked over the surface of LB + 0.3% agar. Swarming mutants (as super swarming subpopulations) were counted per streak after 20 and 36 h of incubation at 30 °C. (**D**) Effect of *bglG* expression on PPD^+^ mutations in the presence of salicin. A total of 10^8^ cells of strains Δ*bglB* and Δ*bglB*_Ptet-G were individually applied to M9 + PPD + salicin (0.5%) plates. (**E**) Effect of *bglG* expression on Glp^+^ mutations in the presence of salicin. A total of 2 × 10^7^ cells of strains Δ*crp* and Δ*crp*_Ptet-G were individually applied to M9 + glycerol + salicin (0.5%) plates. (**F**) Effect of *bglG* expression on swarming mutations in the presence of salicin. A total of 2 × 2 μL of cell suspensions with an OD_600_ of 1 were streaked over the surface of LB+ salicin + 0.3% agar.

We next examined the effects of *bglG* expression on IS5 insertional mutations of the *fucAO* operon [14], the *glpFK* operon [32] and the *flhDC* operon [10]. Agar plates of M9 + PPD, M9 + glycerol and LB + 0.3% agar (soft agar) were first used for these mutational assays in the absence of a β-glucoside. The results are shown in Figure 4A for activation of the *fucAO* operon, in Figure 4B for activation of the *glpFK* operon, and in Figure 4C for activation of the *flhDC* operon. In all three cases, the increased expression of *bglG* had essentially no effect on any of these three types of IS-mediated operon expressional activation mutations.

Similar mutation assays were performed using the same three types of M9 minimal agar plates, each with 0.5% salicin (that is, M9 + PPD + salicin, M9 + glycerol + salicin and LB + 0.3% agar + salicin). The results are summarized in Figure 4D–F, respectively. For PPD^+^ mutation assays on M9 + PPD + salicin agar plates, two *bglB* deletion strains (Δ*bglB* and Δ*bglB* Ptet-G, instead of wild type and Ptet-G), were used so that the Bgl^+^ mutants could not form colonies on these agar plates. As seen in Figure 4D–F, similar to the results obtained from media lacking a β-glucoside, overexpression of *bglG* did not affect the rates of IS insertion into any one of these three operons in the presence of the aromatic β-glucoside, salicin.

### 2.4. The N-Terminal Nucleic Acid Binding Domain of BglG Is Required for Enhancement of Bgl^+^ Mutation Rates

**Figure 5:**
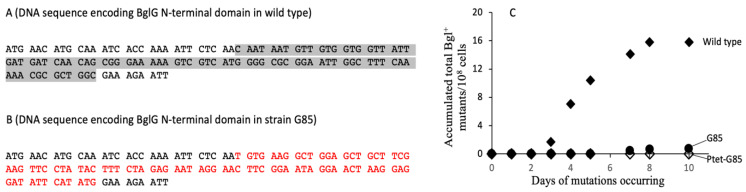
N-terminal domain of BglG is required for enhancement of Bgl^+^ mutations. (**A**) DNA sequence of the portion of *bglG* encoding BglG’s N-terminal domain in the wild type protein. (**B**) DNA sequence of the portion of *bglG* corresponding to the altered N-terminal domain of BglG in strain G85. The 85-bp DNA region in grey (**A**) that encodes 28 N-terminal residues of BglG was replaced by an 85-bp DNA scar (red-faced nucleotides) in (**B**). This 85-bp scar is in frame within the *bglG* gene and has no stop codon. (**C**) Bgl^+^ mutation assays using strain G85 without or with *bglG* overexpression. Strains G85 (with the altered 28 residues at N-terminal residues of the native BglG) and Ptet-G85 (with the intact *bglG* in native *bgl* operon plus P*tet* driving *bglG85* at the *intS* locus of BW25115) were subject to Bgl^+^ mutation assays as described in the legend to Figure 1 and the Section 4. Solid diamonds: wild type *bglG*; solid circles: G85; open diamonds: Ptet-G85.

The N-terminal domain of BglG is believed to be the RNA binding region of the protein, responsible in part for its anti-termination activity, while the C-terminal domain seems to be the BglG dimerization domain [33,34,35], required for conversion of the inactive monomeric protein to the active dimeric anti-terminator protein. To determine if this domain is important for enhancement of Bgl^+^ mutations, 85 nucleotides in this region of the wild type BglG protein (see nucleotides in grey highlight in Figure 5A) were replaced by another 85 nucleotides as indicated in Figure 5B (nucleotides in red). Such nucleotide replacements led to alteration of 28 residues at the N-terminus of BglG. This mutated strain was called G85. To overexpress this mutated gene, another strain, Ptet-G85 was created, in which the modified *bglG* gene, *bglG85*, was driven by P*tet* at the *intS* locus while the native *bglG* gene remained unchanged. 

Figure 5C shows the results obtained when the wild type and altered genes were expressed in the native *bgl* operon. The wild type gene gave rise to the usual Bgl^+^ mutations (solid diamonds), but the mutated gene (that is, strain G85), with the altered N-terminal domain, did not support the appearance of Bgl^+^ mutations (circles). When the altered *bglG* gene was expressed at high levels under the control of P*tet* in the wild type genetic background (that is, Ptet-G85 with the native *bglG* gene present, open diamonds), no Bgl^+^ mutants arose. Clearly, the N-terminally altered *bglG* gene (i.e., *bglG85*) was unable to promote Bgl^+^ mutations. If the *bglG85* gene was overexpressed in the genetic background of the wild type *E. coli* strain (that is, strain Ptet-G85), which still expresses the wild type *bglG* gene, no Bgl^+^ mutants appeared, showing that the N-terminally altered BglG85 competed effectively with the wild type BglG, thereby abolishing its activity. It is therefore concluded that the N-terminal domain, thought to be involved in RNA binding, is essential for promoting the appearance of Bgl^+^ mutations. 

### 2.5. Enhancement of the Bgl^+^ Mutation Rates Depends on BglG Binding to the First Anti-Terminator Site in the Nucleic Acid

**Figure 6:**
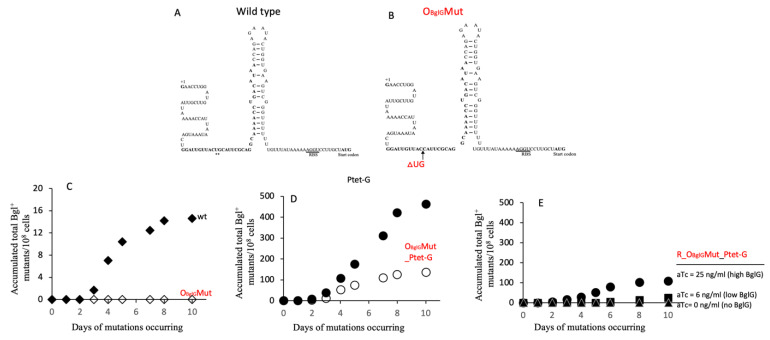
Effects of altering the BglG binding site on the nascent mRNA on the frequency of Bgl^+^ mutations. (**A**) Diagram of the first terminator T1, a Rho-independent hairpin structure. The BglG binding sequences (OBglG), partially overlapping with the hairpin structure, are bolded. (**B**) Diagram of the first terminator with a 2-bp (UG) deletion in the BglG binding site on the mRNA. In both A and B, the transcriptional start site is labeled +1. The RBS is underlined, and the start codon, ATG, of *bglG* is bolded. (**C**) Effect of deleting the “UG” bases in OBglG (referred to as strain “OBglGMut”) on Bgl^+^ mutation frequencies in wild type cells. (**D**) Effect of deleting the “UG” bases in OBglG on Bgl^+^ mutations in Ptet-G cells (that is, strain OBglGMut_Ptet-G). (**E**) Effects of various BglG levels on Bgl^+^ mutation frequencies in OBglGMut cells (that is, strain R_ OBglGMut_Ptet-G) with an altered BglG binding site in the DNA and the nascent mRNA.

The results presented in Figure 6 suggest that binding of BglG to the mRNA is required for enhancement of Bgl^+^ mutations. The two BglG binding sites in the mRNA (one preceding the bglG gene and one following it) each involves part of the terminator structure and part of the upstream region (see the bolded sequence of Figure 6A for the BglG binding site on the first terminator) [36]. The latter was altered by deleting two base pairs in the first antiterminator region of the DNA (and hence the transcribed mRNA). This mutant was called OBglGMut (Figure 6B). When this mutant mRNA was present in a single copy, no Bgl^+^ mutations arose (Figure 6C). However, when using this mutant strain, and a second copy of bglG was expressed under the control of Ptet, some residual mutational activity was observed. However, the number of Bgl^+^ mutations arising was much lower than the control experiment with the wild type mRNA (Figure 6D).

To see if varying amounts of BglG influence the frequency of Bgl^+^ mutations arising in the mutant strain OBglGMut, we created strain R_ OBglGMut_Ptet-G, in which *bglG* expression is repressed by TetR, and BglG levels are proportional to the inducer (that is, anhydrotetracycline, aTc) concentrations. When the aTc was absent, no Bgl^+^ mutations were observed. When 6 ng/mL, and then 25 ng/mL of aTc were present, increasing numbers of Bgl^+^ mutations arose (Figure 6E). However, the increased mutation levels were significantly lower than those obtained using strain R_Ptet-G, that is, the same as strain R_OBglGMut_Ptet-G except for the presence of the native *bglG* (Figure 2B,E).

These experiments show that although the 2 bp deletion in the mRNA BglG binding site diminished the effects of BglG on promotion of Bgl^+^ mutations, it did not eliminate them altogether. Clearly, this suggests that this mutant mRNA can still bind BglG weakly (as observed when *bglG* is expressed at higher levels), but with a larger binding constant (lower affinity). It should be noted that these experiments do not distinguish between BglG binding to the mRNA or to the corresponding region in the encoding DNA, but so far, no evidence suggests that BglG binds to the DNA. Thus, we favor the idea that binding of BglG to the mRNA mediates its effect on the appearance of Bgl^+^ mutants. This may be due to formation of a DNA:RNA hybrid within the transcription bubble, but the binding of BglG to the DNA or the RNA represent two possibilities that cannot be distinguished by the experiments performed to date.

### 2.6. Promoter-Dependent Overexpression of BglG Promotes Residual Bgl Operon Expression

**Figure 7:**
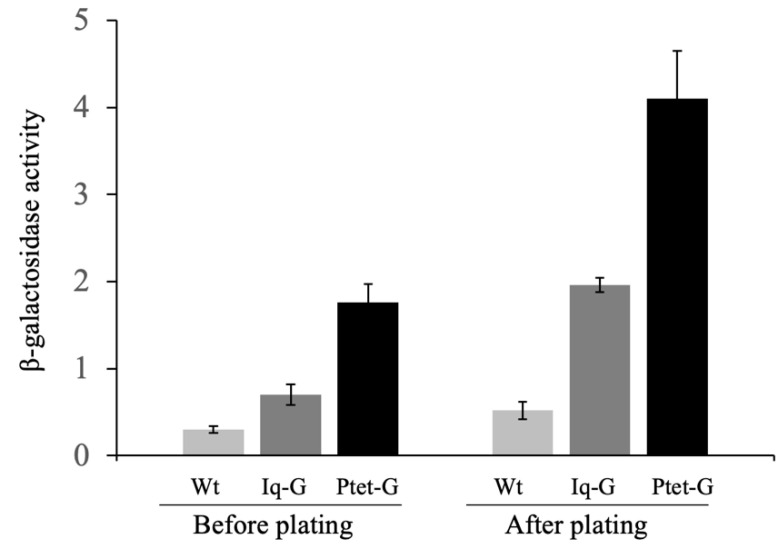
Effects of BglG levels on residual *bgl* operon transcriptional activities in cells before plating and after plating onto M9 + salicin agar plates. The wild-type, Iq-G and Ptet-G cells (all carrying the operon *lacZ* reporter, P*bgl*-*bglG*-*lacZ*, chromosomally integrated at the *lacZ* site) were cultured in LB for 8 h, washed 2x with M9 salts and diluted to OD_600_ of 0.1 before plating. The operon activities before plating, as indicated by β-galactosidase activities, were measured, based on the operon *lacZ* reporter. An amount of 200 μL of the cell suspensions were applied onto M9 + salicin agar plates that were incubated at 30 °C. After 1.5 days of incubation, the cells were washed off the plates using an M9 salts solution, and the cell suspensions were assayed for β-galactosidase activities (i.e., operon activity after plating) (see Methods). β-galactosidase activities were measured following expression of *bglG* under the control of the native *bgl* operon promoter, or after overexpression of *bglG* using either the *lacIq* promoter or the stronger P*tet* promoter. Measurements were made either before plating (left) or after plating (right) as indicated.

Regardless of which promoter drives *bglG* expression, all of our test strains were incapable of growing on β-glucoside-containing plates (Figure S2) or in minimal liquid medium (Figure S3). To see if increased BglG levels had any effects on residual operon activities, using a *bgl* operon-*lacZ* transcriptional fusion reporter, we measured operon expression levels in those three strains (wt, Iq-G and Ptet-G) used for Bgl^+^ mutation assays (see Figure 1). β-Galactosidase activities were first quantitated in LB liquid medium (activities before plating with no β-glucoside) following various levels of expression of *bglG* under the control of these three promoters (left panel of Figure 7). As expected, the Native *bgl* operon promoter gave an extremely low level of expression (0.3 Miller units) while the *lacIq* and P*tet* promoters gave successively more expression (about two-fold increase for Iq-G and six-fold increase for Ptet-G).

Next, these three strains were plated on M9-salicin agar plates exactly as was done in the experiments shown in Figure 1 and Figure 5. After incubation at 30 °C for 36 h (no observed Bgl^+^ mutations occurring during this period), cells were washed off these plates, and β-galactosidase activities (activities after plating) were measured. As seen in Figure 7 (right panel), the *bgl* operon activities increased about two-fold for each strain tested compared to LB medium, indicating that the presence of salicin further elevated residual operon activities. When *bglG* was driven by *lacIq* or P*tet*, the operon activities increased four-fold or eight-fold compared to the corresponding activities after plating, respectively. Please note that this increased residual expression of the *bgl* operon did not allow detectable cell growth with a β-glucoside as the sole carbon source.

## 3. Discussion

In this report, we have provided evidence that expression of the *bglG* gene, encoding a well characterized transcriptional anti-terminator, BglG, exerts a stimulatory effect on the rates of *bgl* operon-activating IS element insertions into the upstream region of the operon, revealing a new function of the BglG protein. BglG exerts its antitermination activity by binding to two known sites in the messenger RNA, one preceding the *bglG* gene and one following it [37,38]. The discovery that BglG exerts an effect on the rates of IS insertions into the *bgl* operon upstream region is novel, but the detailed mechanism is unknown. We suggest that it acts by binding to the mRNA rather than the DNA, only because its binding to the DNA has not been demonstrated [36].

By inactivating BglG function and adding an internal stop codon, we have shown that the presence of the active BglG protein is essential for the appearance of IS insertional mutations upstream of the *bgl* promoter region. The higher levels of BglG led to greater frequencies of insertional mutations. To exert its effect on IS insertion, the N-terminal nucleic acid binding domain of the BglG protein was required as is the first BglG binding site in the nucleic acid, upstream of the *bglG* gene. When the BglG binding site (partially overlapping the first terminator in the nascent mRNA) was altered by deletion of two nucleotides, its effects on Bgl^+^ mutations were abolished if *bglG* was present in single copy. Moreover, it was significantly reduced when *bglG* was overexpressed. Together these results demonstrate that to activate and subsequently increase Bgl^+^ mutations, the BglG protein must bind to the nascent *bgl* mRNA (or to the corresponding region of the DNA) using its N-terminal domain.

Measurement of operon activity showed that increased *bglG* expression leads to marginally increased transcription of the *bgl* operon. This resulted in an increased number of transcription bubbles near the upstream H-NS binding site [39], thereby elevating negative super-helicity of the upstream DNA that should likely contribute to the release of H-NS from the *bgl* promoter due to DNA conformation changes. Occasional dissociation of H-NS from the upstream region may be sufficient to unmask the target recognition sites for IS insertion, thereby enhancing mutation rates.

For specific DNA target sites of transposable genetic elements, a strong preference has been observed for some specific DNA regions such as intergenic regions [40,41] and AT-rich sequences [40,42]. Several non-canonical DNA structures have been identified such as melted bubbles, Z-DNA, H-DNA and G quadruplexes in both prokaryotic and eukaryotic genomes [43,44,45,46,47]. One major representative of DNA structures is the SIDD (superhelical stress-induced DNA duplex destabilization) structures that are preferentially present in regulatory regions [48,49] and specifically promote insertion of IS elements [16]. A strong SIDD structure is present upstream of the *bgl* promoter [16], and the majority of IS insertional mutations occur in this SIDD region [22,50,51]. This same structural feature in the DNA (that is, a SIDD structure) is also associated with environmental stress-induced IS insertions in other target genes or operons such as *glpFK*, *flhDC*, *fucAO* and *nfsB* [16]. Altering the predicted stability of a SIDD structure substantially affects IS insertional rates at their hotspots [16]. Although the precise binding site of H-NS within the *bgl* promoter region is unknown, reported evidence has shown it is embedded within or near the SIDD structure [39]. H-NS is the primary silencer of the *bgl* operon [29,30,39], and its gene is the only one whose deletion or inactivation results in a Bgl^+^ phenotype in wild type *E. coli* cells. Preliminary observations indicate that H-NS inhibited IS insertion in the *bgl* system It is conceivable that H-NS binding to or release from its operator sequences within the SIDD greatly affects the size and stability of the SIDD ssDNA bubble, thereby regulating IS insertional rates. Therefore, any agent or environmental condition that influences cytoplasmic concentrations of H-NS may provide additional possibilities for the regulation of mutation rates.

A further complication involves increases in insertional mutation rates that are dependent on the presence of a beta-glucoside. It is this feature that renders the process responsive to environmental conditions. In fact, we have demonstrated that BglG, either in the presence or the absence of a beta-glucoside, does not influence IS-dependent activation of three other operons (the *glpFK* operon, the *fucAO* operon and the *flhDC* operon) known to be activated by IS insertions. The fact that the activation of these three operons was not altered under the conditions that activated the *bgl* operon led us to believe that the effects of BglG levels on mutation rates are operon specific. This does not, however, eliminate the possibility that BglG influences other events outside of the *bglGFB* operon. This is clearly an open question.

**Figure 8:**
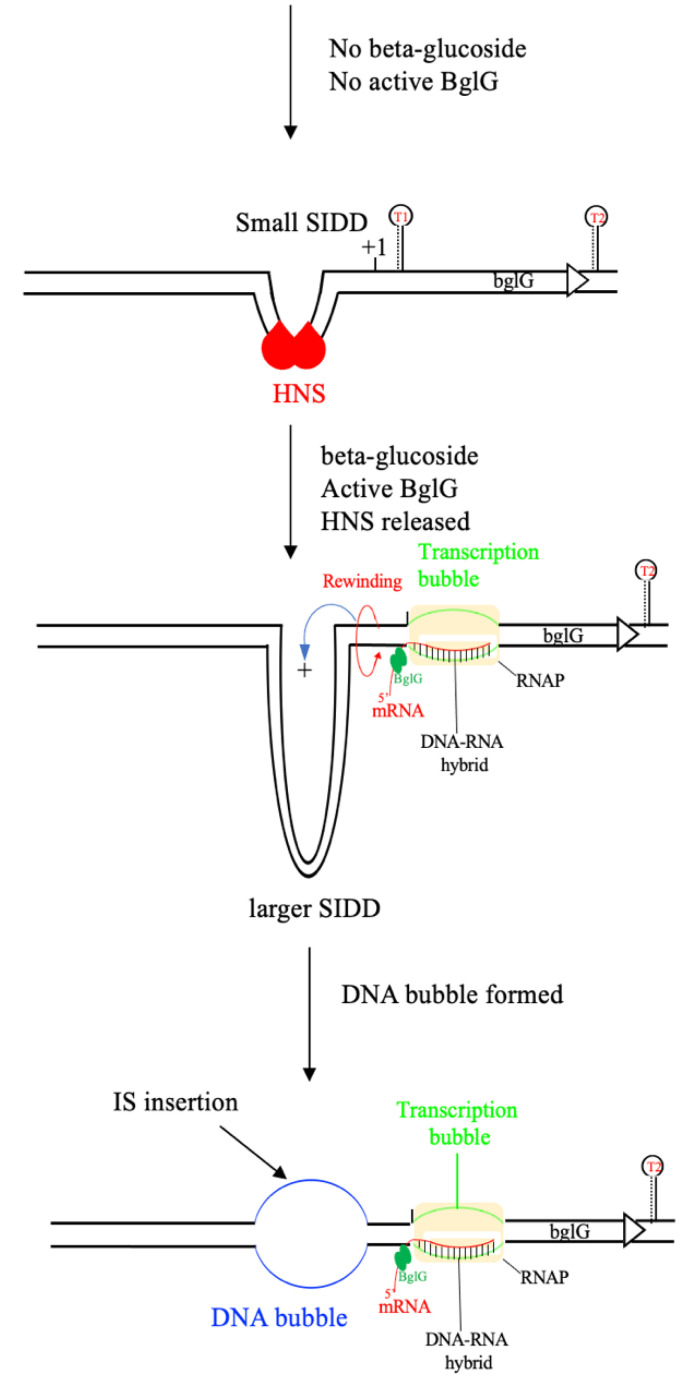
A proposed model for BglG-activated IS insertional Bgl^+^ mutations. In the absence of β-glucoside, no BglG is produced, or if any is produced, BglG would be in the inactivated form. Meanwhile, H-NS binds tightly to the SIDD structure, thereby minimizing the SIDD ssDNA bubble size, blocking both transcription and IS insertion. Therefore, no Bgl+ mutations occur in the absence of a β-glucoside. In the presence of a β-glucoside, the residual BglG is activated and then binds to nascent mRNA that stabilizes the transcription bubble by forming a DNA-RNA hybrid. A stabilized transcription bubble and DNA rewinding lead to a conformational change of the upstream DNA, including the SIDD region, causing the (occasional) release of H-NS from the SIDD. Without H-NS binding, the SIDD structure becomes stronger and larger, and, therefore, it is easier to form a single-stranded DNA bubble. The presence of larger ssDNA bubbles facilitates the insertion of IS elements. In the presence of a β-glucoside, higher levels of activated BglG protein (such as in strains Ptet-G and Iq-G) result in more stabilized transcription bubbles and DNA rewinding, thereby producing one or more larger single-stranded DNA bubbles in the SIDD region, thus promoting IS insertion.

Based on the observations described above, a possible mechanism through which BglG activates insertions of IS elements to the *bgl* operon regulatory region is summarized in Figure 8. 

In the absence of a β-glucoside, the *bgl* operon is silent due to the strong repression by H-NS, and no BglG is produced (or if any BglG is produced, it is in an inactivated state). Meanwhile, H-NS binds tightly to the upstream SIDD region that is important for IS insertion, thereby abolishing the SIDD structure or minimizing its bubble size, thereby blocking IS insertion. Then, no Bgl^+^ mutations occur in the absence of a β-glucoside (a substrate of the *bgl* operon). In the presence of a β-glucoside substrate, the residual level of BglG is activated, and it then binds to nascent mRNA that stabilizes the transcription bubble by forming a BglG/mRNA/DNA ternary complex. A stabilized transcription bubble plus DNA rewinding leads to a conformational change (e.g., increased negative super-helicity) of the upstream DNA, including the SIDD region, causing the (occasional) disassociation of H-NS from the SIDD. Without H-NS binding, the SIDD structure becomes more open, and it is, therefore, easier to form a single-stranded (ss)DNA bubble. The presence of ssDNA bubbles facilitates the insertion of IS elements [16]. In the presence of a β-glucoside, higher levels of activated BglG protein (such as in strains Ptet-G and Iq-G) result in more stabilized transcription bubbles and DNA rewinding, thereby producing larger single-stranded DNA bubbles in the SIDD region to promote IS insertions.

In vitro binding assays showed that BglG failed to bind to the *bgl* DNA [36]. However, it is still possible that BglG binds to the corresponding site in the encoding DNA in vivo in addition to its site in the mRNA, especially when *bglG* is overexpressed. Our preliminary observation showed that when both terminators flanking *bglG,* including the BglG binding sites, were deleted, increased expression of *bglG* still enhanced the frequency of IS insertional Bgl^+^ mutations although at reduced levels relative to the wild type strain Another surprising observation was that we could not successfully construct the P*tet*-*bglG* cassette on a mid-copy number plasmid because only a few clones resulted from each transformation, and all the sequenced clones had mutations either in P*tet* or in the *bglG* gene This indicated that *E. coli* cells dislike too much BglG protein, a nucleic acid binding protein. Therefore, we cannot rule out the possibility that BglG binds to the corresponding DNA region (ssDNA or dsDNA) in vivo with lower affinity than to the mRNA, and this binding interaction (BglG-DNA) might directly influence the IS-activating insertional events. This suggestion is presumed but not established. Further experimentation will be required to determine if stimulation of IS insertion by high level *bglG* expression is due to BglG-mRNA or BglG-DNA complex formation, and if formation of a BglG/mRNA/DNA complex plays a role. 

Another surprising observation resulting from the reported research concerns the appearance of non-IS insertion-type mutations that are also influenced by the level of *bglG* expression. In fact, both IS insertional mutations and non-IS insertional mutations increased in frequency in response to increased BglG concentrations, but the latter increased to a greater extent than the former. Thus, the rates of increase differed, so that at low concentrations of BglG, IS insertional mutations predominated, while at high BglG concentrations, non-IS insertional mutations predominated. Although we anticipate that the two phenomena are related, they clearly also differ, and these two types of mutations may arise independently of each other.

Based on the growth properties using salicin as the carbon source, we could classify IS-independent mutants into several groups: Crp-dependent, Crp-independent, *bglG* overexpression-dependent and *bglG* overexpression-independent. One type of Crp-dependent mutants resulted from a specific internal *hns* gene duplication (Figure S4), but in most cases, the genes affected by most of the non-IS insertional mutations are not known. We do not know how BglG influences the frequencies of these mutational processes. Since these mutations rely on increased *bglG* levels that lead to marginally elevated levels of operon transcription, these non-IS insertional mutations could occur in an energy-dependent process. In agreement with this suggestion, we have shown that these mutations arise only in the presence of a beta-glucoside. It is not clear whether *bglG* expression is influenced by external conditions that would render the process “directed”. Clearly, much remains to be investigated.

## 4. Materials and Methods

### 4.1. Construction of Ptet Driving BglG and LacIq Driving bglG Expression at the IntS Locus on the Chromosome

The region containing the kanamycin (km) resistance gene (*km*^r^), the *rrnB* terminator (*rrnB*T) and the P*tet* promoter (that is, *km*^r^:*rrnB*T:P*tet*) was amplified from pKDT-Ptet [52] using primers Ints-P1 and Ptet-R (Table S2). The *bglG* structural gene was amplified from BW25113 chromosomal DNA using primers bglG-F and IntS-bglG-P2 (Table S2). Note that the 37 nucleotides at the 3′ end of the “*km*^r^:*rrnB*T:P*tet*” fragment are the same as the first 37 nucleotides at the 5′ end of the *bglG* fragment. After gel purification, the *km*^r^:*rrnB*T:P*tet* fragment and the *bglG* gene were fused together by standard fusion PCR using both DNA fragments in equal ratios as templates and IntS-P1 and IntS-bglG-P2 as primers. The resultant fused product, “*km*^r^:*rrnB*T:P*tet*-*bglG*”, was gel purified and then electroporated into BW25113 cells (expressing the λ-Red recombinase) to replace the *intS* gene using the lamada Red method as described in [53]. Several Km resistant colonies were confirmed by PCR and DNA sequencing. One correct clone carrying the “*km*^r^:*rrnB*T:P*tet*-*bglG*” cassette at the *intS* locus was named Ptet-G (Table S1), in which the strong P*tet* promoter constitutively drives *bglG* expression.

In order to express *bglG* at an intermediate level, the constitutive *lacIq* promoter (Iq) was amplified using oligos PIq-Xho and PIq-Kpn from pZSint4 [54], digested with *Xho*I and *Kpn*I, respectively, and it was then ligated into the sites of pKDT [52] digested with the same enzymes, yielding pKDT-Iq. The fragment “*km*^r^:*rrnB*T:Iq” was amplified from pKDT-Iq, and a new *bglG* gene was amplified from the chromosomal DNA. Both fragments were fused together by PCR and integrated into the *intS* locus on the BW25113 chromosome. This yielded strain Iq-G, in which the intermediate strength *lacIq* promoter constitutively drives *bglG* expression. 

To titrate *bglG* expression, a *tetR* expression cassette (that is, a strong constitutive promoter driving *tetR* expression) was transferred from BW-RI [55] into strain Ptet-G, yielding strain R_Ptet-G. In this strain, TetR represses the P*tet* promoter, thereby abolishing *bglG* expression. In the presence of an inducer such as anhydrotracycline (aTc) or chlorotetracycline (cl-Tc), TetR is released from P*tet*, leading to *bglG* expression. The more inducer added, the greater the level of *bglG* expression.

Using P1 transduction, the *bglG* expression cassette, *“km*^r^:*rrnB*T:P*tet*-*bglG*” at the *intS* locus, was transferred into a *crp* deletion strain [8], yielding Δ*crp*_Ptet-G. To make strain Δ*bglB*_Ptet-G, the *bglB* mutation was first transferred to BW25113, yielding strain Δ*bglB*. The *“km*^r^:*rrnB*T:P*tet*-*bglG*” cassette was transferred to Δ*bglB*, yielding strain Δ*bglB*_Ptet-G (Table S1).

### 4.2. Construction of a BglG Mutant with an Altered N-Terminal Domain and Ptet Driving the Modified BglG on the Chromosome

The N-terminal domain is responsible for BglG’s binding to the nascent mRNA [33,34]. To determine if the N-terminal domain is required for BglG stimulation of Bgl^+^ mutations, the region from the 30th to the 114th nucleotides (encoding 28 amino acids; see the grey region in Figure 5A) of *bglG* was replaced by a *km*^r^ gene amplified from pKD4 [53] using oligos bglG.NT-P1 and bglG85-P2 (Table S2). The *km*^r^ gene was then removed by pCP20, and an 85-bp scar (in frame) was left behind (see red-faced region in Figure 5B). Please note that the bglG.NT-P1 oligo was designed with care so that there are no stop codons present in the 85-bp scar after the *km* gene was deleted. This yielded strain G85, in which the modified *bglG* gene (*bglG85*) encodes a new version of BglG with 28 residues changed near the N-terminus of the protein. One codon, AAC, was changed to AAT but both codons encode the same amino acid residue (Figure 5A,B). To express the modified *bglG85* at a greater level, the cassette of P*tet* driving *bglG85* together with a *km*^r^ gene and a terminator (that is, *km*^r^:*rrnB*T:P*tet*-*bgl85*) was assembled by fusion PCR and integrated in the *intS* site of BW25113 using the methods described above for strain Ptet-G. This yielded strain Ptet-G85.

A strain similar to G85 was made in which a stop codon “TAG” was introduced in the 85-bp scar in addition to the altered 28 residues at the N-terminal domain of BglG (Figure S5). To make this strain, the same *km* gene was amplified using bglG.NTstp-P1 and bglG85-P2 (Table S2). The 3′ region of oligo bglG.NTstp-P1 contains the stop codon TAG. The same N-terminal region of *bglG* (grey region of Figure 5A) was replaced by the *km* gene that was subsequently removed by pCP20. A stop codon TAG was then present in the 85-bp scar (Figure S5). The resulting strain, G85stop, has lost the ability to make the BglG protein. To express *bglG* in this strain, the P*tet*-*bglG* cassette (at the *intS* locus) plus or minus the TetR source (a constitutive promoter driving *tetR* at the *attB* locus) was transferred into strain G85stop by P1 transduction, yielding R_G85stop.Ptet-G (with TetR source) and G85stop.Ptet-G (with no TetR source).

### 4.3. Construction of a BglG Binding Site Mutation near the First Terminator, T1

There are two Rho-independent terminators flanking the *bglG* gene in the *bgl* operon, and the first terminator (T1) is located in the 5′ un-translated region of *bglG* (Figure 6A). To complete transcription of the *bgl* operon, the dimerized anti-terminator, BglG, must bind to its operator sequences (the bolded sequences partially overlapped with T1 in Figure 6A) of the nascent mRNA to prevent formation of the first terminator. Houman et al. showed nucleotides UG (+51 and +52 from the transcription start site, +1) are required for BglG binding [36]. To remove these two nucleotides, they were first replaced by the *tetA*-*sacB* cassette that was amplified from the genomic DNA of strain T-SACK [56] using oligos ObglG-tet-F and ObglG-sac-R. Several tetracycline-resistant (Tc^r^) and sucrose-sensitive (Suc^s^) colonies were confirmed by PCR and then sequencing for such a replacement. A 100-bp DNA oligo, ObglG-100 (Table S2), that carries the TG deletion and consists of 50 nucleotides immediately upstream and downstream of the TG was substituted for the *tetA*-*sacB* cassette in the cells of a confirmed Tc^r^ and Suc^s^ clone. The transformants were selected on LB + sucrose (6%, *w*/*v*) + fusaric acid (24 mg/liter) agar plates. Several Suc^r^ and Tc^s^ colonies were confirmed for the replacement by PCR and sequencing. One such clone was named OBglGMut, in which the nucleotides, TG, from the conserved BglG-binding site were deleted. The cassette of P*tet* driving *bglG* was transferred to OBglGMut, yielding strain OBglGMut_Ptet-G. The *tetR* cassette (a constitutive promoter driving *tetR*) was transferred to OBglGMut_Ptet-G, yielding strain R_OBglGMut_Ptet-G (Table S1).

### 4.4. Construction of the Bgl Operon LacZ Reporter

The region carrying the *bgl* promoter and the first gene in the operon, *bglG*, including the 2nd terminator (the 335th nucleotide upstream of the translational start codon of *bglG* to the 9th codon of *bglF*) was amplified from the BW25113 genomic DNA using primers Pbgl-Xho-F/bglG-BamH-R (Table S2). A stop codon, TAA, was added to the end of *bglF*’s 9th codon. The PCR products, which carried the *bglGFB* promoter, the first terminator, *bglG*, the *bglG*/*bglF* intergenic region (containing the 2nd terminator) and the first 9 codons of *bglF* plus a stop codon at the 3′ end, were digested with *XhoI* and *BamHI* and then inserted into the same sites of the plasmid pKDT [52], yielding the plasmid pKDT_P*bgl-bglG*. Present in this plasmid, the “*km*:*rrnB*T:P*bgl-bglG*” cassette was amplified using primers Pbgl-Z1/bglG-Z2 and then integrated into the chromosome of default strain EQ42 [52] to replace the region containing *lacI* and the entire *lacZ* promoter. After being confirmed by colony PCR and DNA sequencing, the P*bgl-bglG*-*lacZ* transcriptional fusion was transferred to BW25113, Iq-G and Ptet-G by P1 phage transduction, yielding strains BW25113_Z, Iq-G-Z, and Ptet-G_Z, respectively (Table S1).

### 4.5. Bgl^+^ Mutation Assays

Bgl^+^ mutation assays were performed on minimal M9 agar plates with 0.5% of a β-glucoside such as salicin or arbutin as the sole carbon source. Strains to be tested (from single fresh colonies) were cultured in LB liquid medium for about 8 h at 30 °C, washed twice using 1x carbon source-free M9 salts (M9) and applied onto plates (2 × 10^7^ cells/plate). The plates were then incubated in a 30 °C incubator and were examined daily for the appearance of Bgl^+^ colonies with each colony representing a new Bgl^+^ mutation. On these β-glucoside minimal agar plates, any colonies appearing by day 2 were considered to be from Bgl^+^ cells initially applied onto the plates. They were therefore subtracted from the subsequent measurements. 

The total numbers of Bgl^−^ cells (background populations) were determined as described previously [8,31]. Briefly, agar plugs (diameter = 1 cm) were removed using a sterile borer from plates to glass tubes containing sterile M9 salts with no carbon source. The cells were washed off by vortexing. The washings were appropriately diluted and then applied onto LB and M9 + salicin plates. The total Bgl^−^ cell population (per agar plug) was determined by subtracting the populations obtained from M9 + salicin plates from the populations obtained from LB plates. The total Bgl^−^ cell population per agar plate (9 cm in diameter) was the total Bgl^−^ population per agar plug multiplied by 81. The frequencies of Bgl^+^ mutations on salicin M9 plates were determined by dividing the total Bgl^+^ populations by the number of Bgl^−^ colonies. 

To determine the effects of other carbon sources on Bgl^+^ mutations, mutation assays were performed on minimal M9 agar plates with 0.25% glycerol or 1% propandiol as the sole carbon source. To determine the total populations, the cells were washed off the minimal M9 agar plates daily, serially diluted and plated onto LB agar plates. To determine Bgl^+^ populations, appropriate dilutions were applied on M9 + salicin agar plates. The frequencies of Bgl^+^ mutations were determined as described above for Bgl^+^ on M9 + salicin agar plates.

### 4.6. Glp^+^ Mutation Assays

Glp^+^ mutation assays were conducted on glycerol M9 minimal agar plates as described in Zhang and Saier [8]. Strains D*crp* and D*crp* Ptet-G were used for the mutation assays. To test the effects of b-glucoside on Glp^+^ mutations, salicin (0.5%) was added together with glycerol on M9 agar.

### 4.7. Swarming Mutation Assays

Using the wild type and Ptet-G strains, the swarming mutation assays for the appearance of hyper-swarming mutants (outgrowing subpopulations from the inoculated cells) were carried out using the method of Barker et al. [57]. Briefly, overnight cell cultures in LB media were washed once with M9 salts and diluted to an OD_600_ of 1.0 prior to use. Two µL of the cell suspensions were streaked across the centers of LB semisolid (0.3% agar) plates (diameter = 9 cm) using a plastic transfer loop. LB and LB + 0.5% salicin media were used. The plates were incubated at 30 °C. The swarming mutants, represented by outgrowths of motile subpopulations from the streaked cells, were counted. The mutation frequency was normalized as outgrowths (mutations) per 9-cm cell streak.

### 4.8. Propanediol (PPD) Growth Mutation Assay

The assay for PPD^+^ mutations was conducted by applying cell suspensions from fresh overnight cultures onto propanediol (1%) ± salicin (0.5%) M9 minimal agar plates (~10^8^ cells/plate). The wild type and Ptet-G strains were used for M9 + salicin plate assays while Δ*bglB* and Δ*bglB* Ptet-G were used for M9 + PPD + salicin plate assays. Growth positive mutations and total populations were determined as described above under “Bgl^+^ mutation assays”.

### 4.9. β-Galactosidase Assays

*E. coli* reporter strains were grown in 4 mL of LB contained in 18 mm diameter glass test tubes with shaking at 30 °C. After 8 h, samples were removed, washed 2x with M9 salts and diluted to an OD_600_ of 0.1. Such cell suspensions (before plating) were subject to measurement of β-galactosidase activities. An amount of 200 μL of the cell suspensions (OD_600_ = 0.1) were applied onto minimal M9 salicin agar plates. After a 36-h incubation at 30 °C, cells were washed off the plates using M9 salts, and the cell suspensions were used for β-galactosidase activity measurements.

To measure β-galactosidase activities, 0.5 mL of Z-buffer containing β-mercaptoethanol (2.7 μL/mL) and SDS (0.005%) were mixed with 0.5 mL of sample and 25 μL of CHCl_3_ in test tubes. The tubes were vortexed twice (each time for 10 s at a constant speed) and incubated in a 37 °C water bath until equilibration. A 0.2 mL aliquot of ONPG substrate (4 mg/mL) was then added to each test tube. When yellow color developed, the reaction was stopped by adding 0.5 mL of 1 M Na_2_CO_3_ followed by vortexing. Reaction mixtures were centrifuged (15k rpm, 3 min), and the absorbance values of the supernatants were measured at 420 nm and 550 nm. A control tube was run in parallel using M9 salts instead of the test sample. β-Galactosidase activity (Miller units) = [(OD_420_ − 1.75 × OD_550_)/(sample volume in mL × time in min × OD_600nm_)] × 1000 [58].

## 5. Conclusions

We here provide evidence that several Insertion Sequence (IS) elements when inserted upstream of the *E. coli bgl* operon promoter represent a novel form of directed mutation. IS insertion is absolutely dependent on the presence of the *bglG* gene product, BglG. Analyses revealed the requirements for this novel function of BglG. Moreover, IS insertion occurs only in the presence of an external aromatic beta-glucoside substrate, thus showing that IS insertion is dependent on environmental conditions. A molecular mechanism for the regulation of this directed mutation is proposed.

## Data Availability

The data presented in this study are available on request from the corresponding author.

## Supplementary Materials

The following supporting information can be downloaded at: https://www.mdpi.com/article/10.3390/ijms23031505/s1.
