# Supplementary material for: Insertion Sequence (IS) Element-Mediated Activating Mutations of the Cryptic Aromatic β-Glucoside Utilization (BglGFB) Operon Are Promoted by the Anti-Terminator Protein (BglG) in Escherichia coli
Source: Int J Mol Sci. 2022 Jan 28;23(3):1505. doi: 10.3390/ijms23031505 (PMC8836124; doi:10.3390/ijms23031505)
Supplement: Supplementary file 1 [file ijms-23-01505-s001.zip › Supplementary Files 12 27 21.pdf]

Supplementary Materials

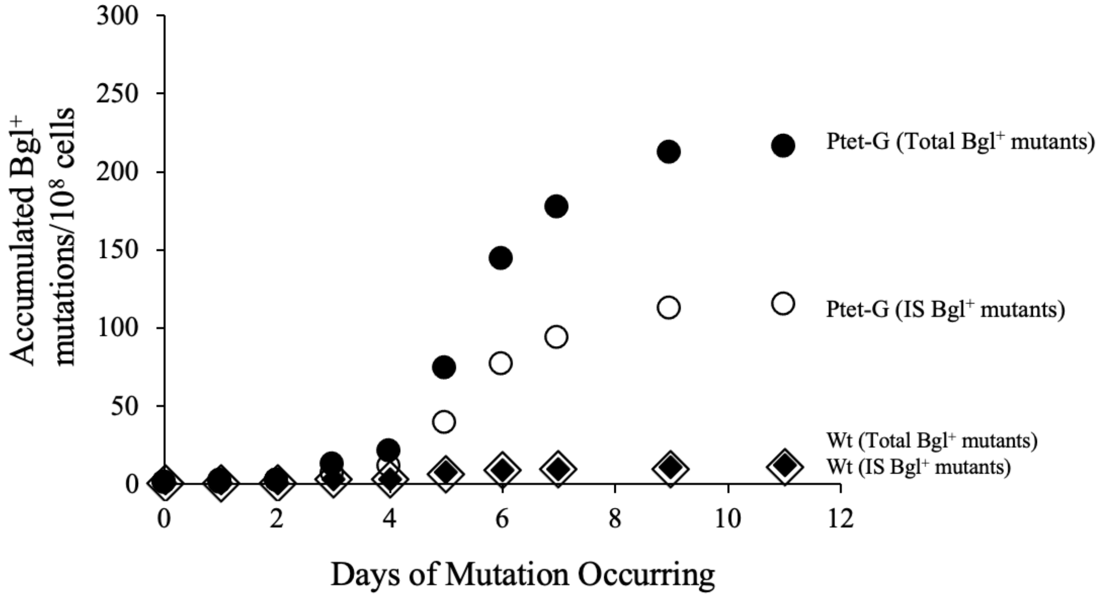

Figure S1. Bgl$^+$ mutations on minimal M9 + arbutin agar plates comparing wild type and Ptet-G. 2 x 10^7 cells were applied onto each minimal M9 agar plate containing 0.5% arbutin as the sole carbon source. The frequencies of total Bgl$^+$ mutations and IS insertional Bgl$^+$ mutations were determined as described in Figure 1 and the Methods section. Solid diamonds: total mutations of wild type; open diamonds: IS insertional mutations of wild type; solid circles: total mutations of Ptet-G; open circles: IS insertional mutations of Ptet-G.

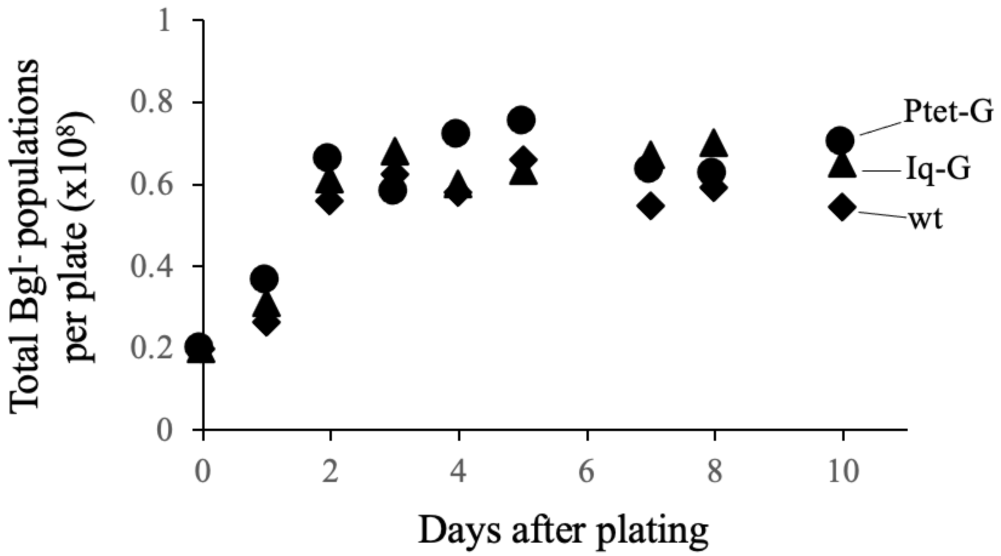

Figure S2. Determination of total Bgl- cell populations on M9 + salicin agar plates. The total Bgl- cell populations of strains wild type, Iq-G and Ptet-G used for Bgl+ mutation assays on M9 + salicin agar plates (see Figure 1) were determined as reported previously (1916241 and 19682247). See the Methods. Some growth (about 1.5 generations) was observed for the Bgl- populations (background populations) in the first two days, and afterwards the populations were relatively constant.

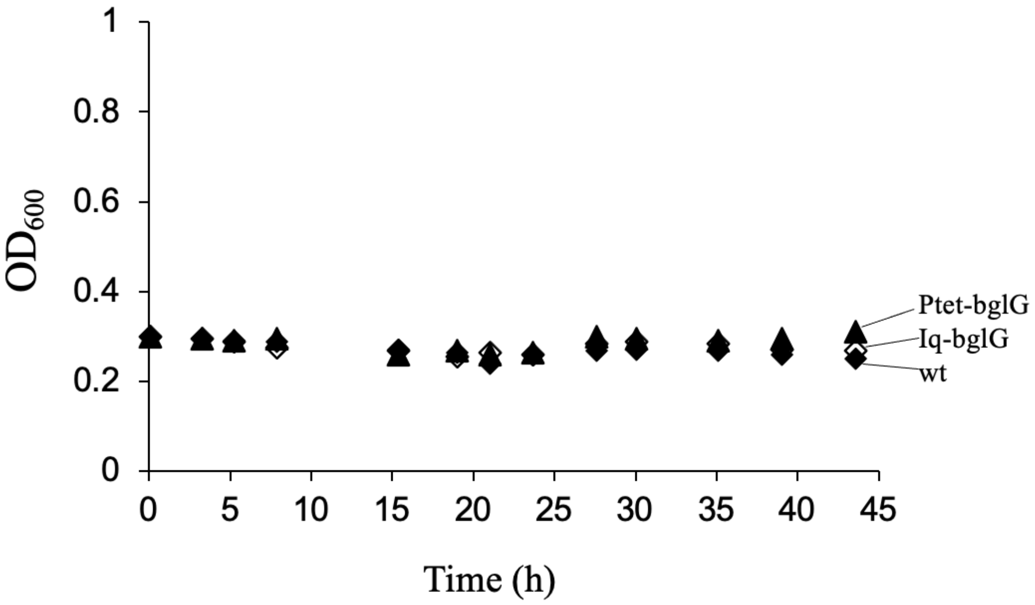

Figure S3. Determination of total populations of strains wild type, Iq-G and Ptet-G in liquid M9 + salicin medium. Test strains (from single fresh colonies) were cultured in LB liquid medium for about 8 h at 30°C, washed twice using M9 salts and diluted into 5ml of liquid M9 + salicin medium (initial OD_{600}=0.3) in glass tubes (18 cm x 1.5 cm). The tubes were rotated (250 rpm) at 30°C, and the optical densities (OD_{600}) were measured over time.

A (Duplication of 12 nucleotides in *hns* of an IS-independent Bgl^{+} mutant)

```
ATG AGC GAA GCA CTT AAA ATT CTG AAC AAC ATC CGT ACT CTT CGT GCG
CAG GCA AGA GAA TGT ACA CTT GAA ACG CTG GAA GAA ATG CTG GAA GAA
ATG CTG GAA AAA TTA GAA GTT GTT GTT AAC GAA CGT CGC GAA GAA GAA
AGC GCG GCT GCT GCT GAA GTT GAA GAG CGC ACT CGT AAA CTG CAG CAA
TAT CGC GAA ATG CTG ATC GCT GAC GGT ATT GAC CCG AAC GAA CTG CTG
AAT AGC CTT GCT GCC GTT AAA TCT GGC ACC AAA GCT AAA CGT GCT CAG
CGT CCG GCA AAA TAT AGC TAC GTT GAC GAA AAC GGC GAA ACT AAA ACC
TGG ACT GGC CAA GGC CGT ACT CCA GCT GTA ATC AAA AAA GCA ATG GAT
GAG CAA GGT AAA TCC CTC GAC GAT TTC CTG ATC AAG CAA TAA
```

B (Duplication of four residues "EMLE" of in HNS)

```
MSEALKILNNIRTLRAQARECTLETLEEMLEEMLEKLEVVVNERREEESAAAAEVEERTRKLQQ
YREMLIADGIDPNELLNSLAAVKSGTKAKRAQRPAKYSYVDENGETKTWTGQGRTPAVIKKAMD
EQGKSLDDFLIKQ
```

Figure S4. Identification of a non-IS insertional Bgl⁺ mutation. A. A duplication of 12 nucleotides in the *hns* gene of an IS-independent Bgl⁺ mutant. The 12 bolded nucleotides in red are the direct repeat of the 12 bolded nucleotides in black. **B**. The 12 nucleotide duplication leads to a duplication of four residues, EMLE, in H-NS. The bolded "EMLE" residues in red are the direct repeat of the bolded residues in black.

A

ATG AAC ATG CAA ATC ACC AAA ATT CTC AAT GTG **TAG** GCT GGA
GCT GCT TCG AAG TTC CTA TAC TTT CTA GAG AAT AGG AAC TTC
GGA ATA GGA ACT AAG GAG GAT ATT CAT ATG GAA AGA ATT

B

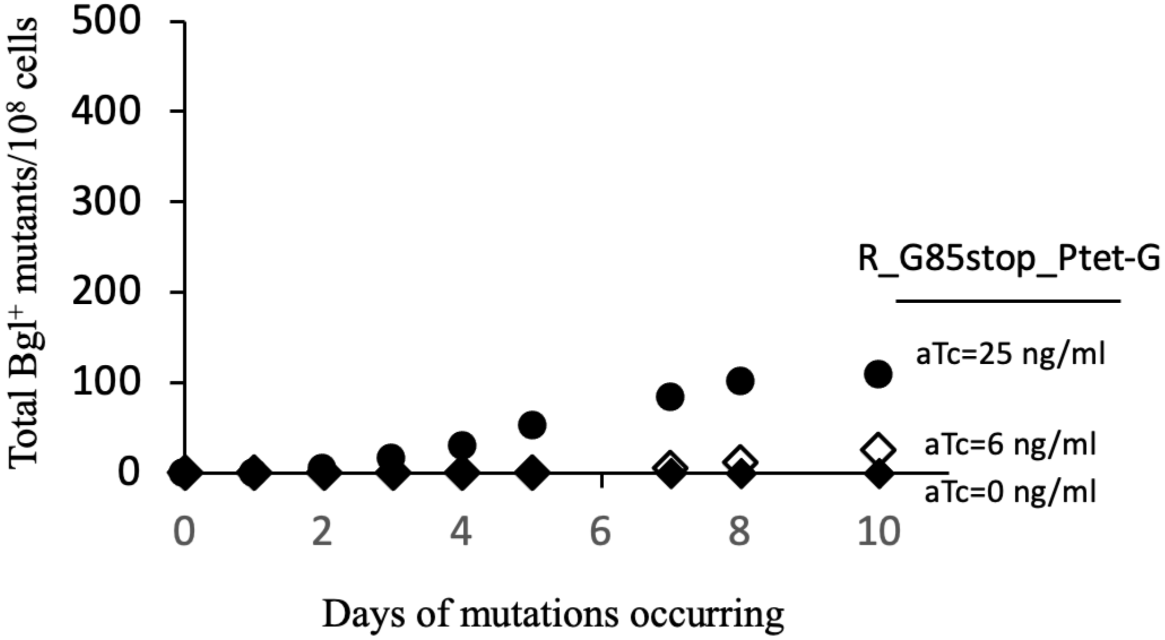

Figure S5. Construction of strain R_G85stop_Ptet-G and Bgl⁺ mutation assay using this strain. A. Construction of a *bglG* knockout mutant G85stop. The DNA sequence of the portion of *bglG* corresponding to the altered N-terminal domain of BglG in strain G85 is shown. The 85-bp DNA region in grey (Figure 5A) was replaced by an 85-bp DNA scar (red-faced nucleotides in Figure 5S). This 85-bp scar carries a stop codon, TAG (yellow highlight), so that no intact BglG protein is produced in this strain. This strain was named G85stop. The constitutively expressed *tetR* and the P*tet-bglG* cassette were transferred to G85stop, yielding strain R_G85stop_Ptet-G. **B.** Bgl⁺ mutation assay using strain R_G85stop_Ptet-G. Mutation assays were conducted on the agar plates of M9 + salicin + aTc (0 to 25 ng/ml aTc) as described in Figure 1 and the Methods section.

Table S1. Strains and plasmids used in this study

Strains or plasmids	Genotype or description	Reference or source
**Strains**		
BW25113	Wild type, *lacI*^{q} *rrnB*$_{T14}$ Δ*lacZ*$_{WJ}$ Δ*hsdR*514 Δ*araBAD*$_{AH33}$ Δ*rhaBAD*$_{LD78}$	PMID 10829079
Ptet-G	P*tet* driving *bglG* at the *intS* locus in BW25113	This study
Iq-G	*lacIq* driving *bglG* at the *intS* locus in BW25113	This study
BW-RI	BW25113 constitutively expressing TetR	PMID 17713988
R_Ptet-G	Ptet-G constitutively expressing TetR	This study
Δ*crp*	Deletion of *crp* in BW25113	PMID 19682247
Δ*crp*_Ptet-G	P*tet* driving *bglG* at the *intS* locus in Δ*crp*	This study
Δ*bglB*	Deletion of *bglB* in BW25113	This study
Δ*bglB*_Ptet-G	P*tet* driving *bglG* at the *intS* locus in Δ*bglB*	This study
G85	Altered 28 residues at the N-terminus of BglG in BW25113	This study
G85stop	One stop codon introduced at the N-terminus of BglG in G85	This study
G85stop_Ptet-G	P*tet* driving *bglG* at the *intS* locus in G85stop	This study
R_G85stop_Ptet-G	G85stop_Ptet-G constitutively expressing TetR	This study
Ptet-G85	P*tet* driving *bglG85* at the *intS* locus in BW25113	This study
T-SACK	Template for the *tetA-sacB* cassette	PMID 24203710
O$_{BglG}$Mut	BglG binding site mutation in terminator T1 of the *bgl* operon	This study
O$_{BglG}$Mut_Ptet-G	P*tet* driving *bglG* at the *intS* locus in O$_{BglG}$Mut	This study
R_O$_{BglG}$Mut_Ptet-G	O$_{BglG}$Mut_Ptet-G constitutively expressing TetR	This study
EQ42	Seamless deletion of *lacY*	PMID 20064380
BW25113_Z	BW25113 carrying the *bgl* operon reporter P*bgl-bglG-lacZ* at the *lac* locus	This study
Ptet-G_Z	Ptet-G carrying the *bgl* operon reporter P*bgl-bglG-lacZ* at the *lac* locus	This study
Iq-G_Z	Iq-G carrying the *bgl* operon reporter P*bgl-bglG-lacZ* at the *lac* locus	This study
**Plasmids**		
pKDT	A *rrnB* terminator (*rrnB*T) in pKD13	PMID 20064380
pKDT_Ptet	P*tet* cloned downstream of the *rrnB* terminator in pKDT	PMID 20064380
pZSint4	A constitutive promoter driving t*etR*	PMID 9092630
pKDT_Iq	*lacIq* cloned downstream of the *rrnB* terminator in pKDT	This study
pKD4	Km resistance and use for chromosomal integration	PMID 10829079
pKDT_P*bgl-bglG*	The *bgl* promoter driving *bglG* in pKDT	This study

Table S2. Oligonucleotides used in this study

Name	Sequence	Use
intS-P1	agatttacagttcgtcatggttcgcttcagatcgttgacagccgcactccatgtgtaggc tggagctgcttc	Amplification of *km*^{r}:*rrnB*T:P*tet* from plasmid pKDT-P*tet* and fusion of *km*^{r}:*rrnB*T:P*tet* to *bglG*
Ptet-R	gaattttggtgatttgcatgttcatggtacctttctcctctttaatgaattc	Amplification of *km*^{r}:*rrnB*T:P*tet* from plasmid pKDT-P*tet*
bglG-F	gaaaggtaccatgaacatgcaaatcaccaaaattctc	Amplification of *bglG* from BW25113 chromosome
intS-bglG-P2	gatagttgttaaggtcgctcactccaccttctcatcaagccagtccgcccttcagtgttct ttgcgcacgcgctc	Amplification of *bglG* from BW25113 chromosome and fusion of *km*^{r}:*rrnB*T:Ptet *to bglG*
intS-ver-R2	aaaggaatgaagtctatcatccaagtc	Verification of integration of *km*^{r}:*rrnB*T:Ptet-*bglG* into the *intS* site
Pbgl-Xho-F	atactcgagtggcgatgagctggataaactgctg	Amplification of *bgl* operon fragment for *lacZ* fusions
bglG-BamH-R	ttaggatccttagactatttttctggctaactccgtc	Amplification of *bgl* operon fragment for *lacZ* fusions
Pbgl-Z1	gcatttacgttgacaccatcgaatggcgcaaaacctttcgcggtatgtgtaggctggag ctgcttc	Amplification of *km*:*rrnB*T:P*bgl*-*bglG* from plasmid pKDT_P*bgl*-*bglG*
bglG-Z2	cgacggccagtgaatccgtaatcatggtcatagctgtttcctgtgtgaaattagactattt ttctggctaactcc	Amplification of *km*:*rrnB*T:P*bgl*-*bglG* from plasmid pKDT*_Pbgl*-*bglG*
Iq-Xho-F	atactcgagtgatgtcattctggtgaagaag	Cloning *lacIq* into pKDT
Iq-Kpn-R	catggtacccaccctgaattgactctc	Cloning *lacIq* into pKDT
lacIq-R	gagaattttggtgatttgcatgttcatattcaccaccctgaattgactctcttccgggc	Amplification of *km*^{r}:*rrnB*T:Iq from plasmid pKDT-Iq
Iq.bglG-F	gagtcaattcagggtggtgaatatgaacatgcaaatcaccaaaattctcaac	Amplification of *bglG* from BW25113 chromosome
bglG.NT-P1	gtttataaaaaaggtccttgctatgaacatgcaaatcaccaaaattctcaatgtgaaggc tggagctgcttcg	Replacing BglG N-terminus with a *km*^{r} gene plus no stop codon
bglG85-P2	catgactgctcaaggcatactctttttctattccacttgagttaattctttccatatgaatatc ctccttag	Replacing BglG N-terminus with a *km*^{r} gene
bglG.NTstp-P1	gtttataaaaaaggtccttgctatgaacatgcaaatcaccaaaattctcaatgtgtaggct ggagctgcttcg	Replacing BglG N-terminus with a *km*^{r} gene plus a stop codon
bglG85-Rn	cacattgagaattttggtgatttgcatgttcatggtacctttctc	P*tet* driving *bglG85* at the *intS* locus
bglG85-Fn	gaaaggtaccatgaacatgcaaatcaccaaaattctcaatgtg	P*tet* driving *bglG85* at the *intS* locus
ObglG-tet-F	ccgaacctggatgttcgttataaaaaccattaataaatgactggattgttactcctaatttt gttgacactctatc	Amplification of the *tetA*-*sacB* cassette from strain T-SACK
ObglG-sac-R	aacccgacttcaccagtattctctggttatgtcaggttttgcctgcgaatg atcaaagggaaaactgtccatatgc	Amplification of the *tetA*-*sacB* cassette from strain T-SACK
ObglG-100	gaacctggatgttcgttataaaaaccattaataaatgactggattgttaccattcgcagg caaaacctgacataaccagagaatactggtgaagtcgggt	100 bp oligo carrying the TG deletion used for replacing the *tetA*-*sacB* cassette
